# Supplementary material for: GSK3 inhibition rescues growth and telomere dysfunction in dyskeratosis congenita iPSC-derived type II alveolar epithelial cells
Source: eLife. 2022 May 13;11:e64430. doi: 10.7554/eLife.64430 (PMC9200405; doi:10.7554/eLife.64430)
Supplement: Figure 1—figure supplement 1—source data 1. — Figure 1—figure supplement 1B-SourceData-2020-07-17-FigureS1-NdeIGeno-CroppingLabeled.tiff – Genotyping agarose gel showing area that was cropped for the Figure 1—figure supplement 1B. Figure 1—figure supplement 1B-SourceData-2020-07-17-FigureS1-NdeIGeno.tiff – Raw agarose gel image for Figure 1—figure supplement 1B. Figure 1—figure supplement 1C-SourceData-DKC1A386T-34.2DA9_B05_045.ab1 – Sanger sequencing file for Figure 1—figure supplement 1C of BU3 NGST DKC1 A386T mutant iPS line. Figure 1—figure supplement 1C-SourceData-WT-33.3EF6_A05_047.ab1 – Sanger sequencing file for Figure 1—figure supplement 1C of BU3 NGST wild type (WT) control iPS line. Figure 1—figure supplement 1E-SourceData-DKC1A386T-KaryotypeImageCLG-35870.pdf – Image of a representative karyotype of BU3 NGST DKC1 A386T iPS line. Figure 1—figure supplement 1E-SourceData-DKC1A386T-REPORTCLG-35870.pdf – Report of karyotype analysis for BU3 NGST DKC1 A386T iPS line. Figure 1—figure supplement 1E-SourceData-WT-KaryotypeImageCLG-35869.pdf – Image of a representative karyotype of BU3 NGST WT control iPS line. Figure 1—figure supplement 1E-SourceData-WT-REPORTCLG-35869.pdf – Report of karyotype analysis for BU3 NGST WT control iPS line. [file elife-64430-fig1-figsupp1-data1.zip › Figure1_FigureSupplement1_SourceData/Figure1-FigureSupplement1E-SourceData-DKC1A386T-REPORTCLG-35870.pdf]

Cell Line GENETICS®

Cell Line Characterization

Cell Line ID: BU3 NGST 2DA9
Passage #: 36
Specimen Type: Human iPSC Culture
Indication for Study: Routine Culture QC

Lab #: CLG-35870
PI: Brad Johnson
Contact Person: Rafael Fernandez
Email: rfern@pennmedicine.upenn.edu
Address:
University of Pennsylvania
Room 406
422 Curie Boulevard
Philadelphia, Pennsylvania 19104
Additional copies sent to:

Test Code: 100
Account #: NA
PO #: 4232892

Date Received: 4/23/2019
Date Reported: 5/6/2019
Time in Culture: 1 Day

Banding Technique: GTL
Band Resolution: Fair
Metaphases Counted: 20 Analyzed: 7 Karyotyped: 2

RESULTS: 46,XY[20] Apparently NORMAL Human Male Karyotype

Non-clonal Aberrations: None

INTERPRETATION:

Cytogenetic analysis was performed on twenty G-banded metaphase cells from human cell line BU3 NGST 2DA9 p36, and all twenty cells demonstrated an apparently normal male karyotype.
