# Supplementary figures and images for: GSK3 inhibition rescues growth and telomere dysfunction in dyskeratosis congenita iPSC-derived type II alveolar epithelial cells
Source: eLife. 2022 May 13;11:e64430. doi: 10.7554/eLife.64430 (PMC9200405; doi:10.7554/eLife.64430)

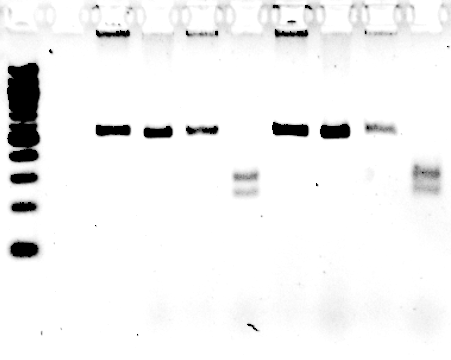

Supplement: Figure 1—figure supplement 1—source data 1. — Figure 1—figure supplement 1B-SourceData-2020-07-17-FigureS1-NdeIGeno-CroppingLabeled.tiff – Genotyping agarose gel showing area that was cropped for the Figure 1—figure supplement 1B. Figure 1—figure supplement 1B-SourceData-2020-07-17-FigureS1-NdeIGeno.tiff – Raw agarose gel image for Figure 1—figure supplement 1B. Figure 1—figure supplement 1C-SourceData-DKC1A386T-34.2DA9_B05_045.ab1 – Sanger sequencing file for Figure 1—figure supplement 1C of BU3 NGST DKC1 A386T mutant iPS line. Figure 1—figure supplement 1C-SourceData-WT-33.3EF6_A05_047.ab1 – Sanger sequencing file for Figure 1—figure supplement 1C of BU3 NGST wild type (WT) control iPS line. Figure 1—figure supplement 1E-SourceData-DKC1A386T-KaryotypeImageCLG-35870.pdf – Image of a representative karyotype of BU3 NGST DKC1 A386T iPS line. Figure 1—figure supplement 1E-SourceData-DKC1A386T-REPORTCLG-35870.pdf – Report of karyotype analysis for BU3 NGST DKC1 A386T iPS line. Figure 1—figure supplement 1E-SourceData-WT-KaryotypeImageCLG-35869.pdf – Image of a representative karyotype of BU3 NGST WT control iPS line. Figure 1—figure supplement 1E-SourceData-WT-REPORTCLG-35869.pdf – Report of karyotype analysis for BU3 NGST WT control iPS line. [file elife-64430-fig1-figsupp1-data1.zip › Figure1_FigureSupplement1_SourceData/Figure1-FigureSupplement1B-SourceData-2020-07-17-FigureS1-NdeIGeno.tiff]

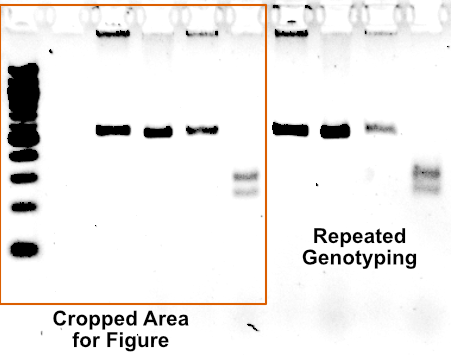

Supplement: Figure 1—figure supplement 1—source data 1. — Figure 1—figure supplement 1B-SourceData-2020-07-17-FigureS1-NdeIGeno-CroppingLabeled.tiff – Genotyping agarose gel showing area that was cropped for the Figure 1—figure supplement 1B. Figure 1—figure supplement 1B-SourceData-2020-07-17-FigureS1-NdeIGeno.tiff – Raw agarose gel image for Figure 1—figure supplement 1B. Figure 1—figure supplement 1C-SourceData-DKC1A386T-34.2DA9_B05_045.ab1 – Sanger sequencing file for Figure 1—figure supplement 1C of BU3 NGST DKC1 A386T mutant iPS line. Figure 1—figure supplement 1C-SourceData-WT-33.3EF6_A05_047.ab1 – Sanger sequencing file for Figure 1—figure supplement 1C of BU3 NGST wild type (WT) control iPS line. Figure 1—figure supplement 1E-SourceData-DKC1A386T-KaryotypeImageCLG-35870.pdf – Image of a representative karyotype of BU3 NGST DKC1 A386T iPS line. Figure 1—figure supplement 1E-SourceData-DKC1A386T-REPORTCLG-35870.pdf – Report of karyotype analysis for BU3 NGST DKC1 A386T iPS line. Figure 1—figure supplement 1E-SourceData-WT-KaryotypeImageCLG-35869.pdf – Image of a representative karyotype of BU3 NGST WT control iPS line. Figure 1—figure supplement 1E-SourceData-WT-REPORTCLG-35869.pdf – Report of karyotype analysis for BU3 NGST WT control iPS line. [file elife-64430-fig1-figsupp1-data1.zip › Figure1_FigureSupplement1_SourceData/Figure1-FigureSupplement1B-SourceData-2020-07-17-FigureS1-NdeIGeno-CroppingLabeled.tiff]

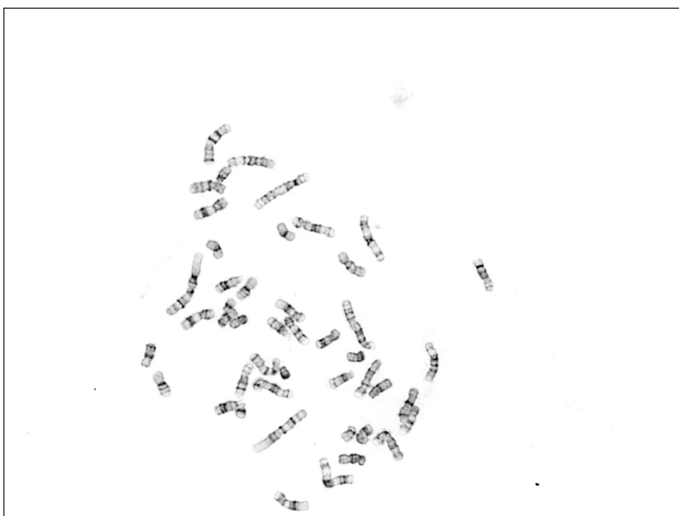

Result: 46,XY

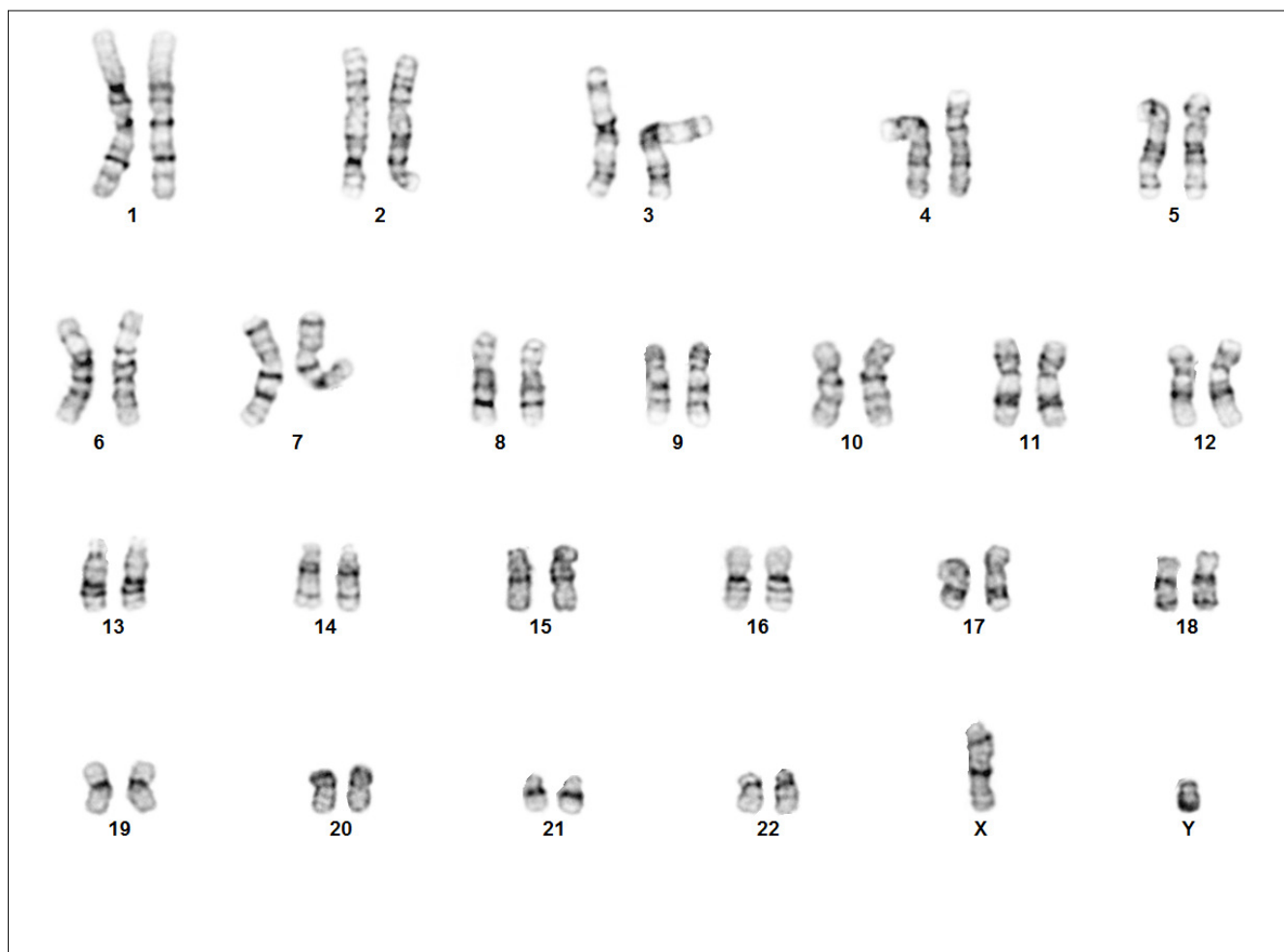

Supplement: Figure 1—figure supplement 1—source data 1. — Figure 1—figure supplement 1B-SourceData-2020-07-17-FigureS1-NdeIGeno-CroppingLabeled.tiff – Genotyping agarose gel showing area that was cropped for the Figure 1—figure supplement 1B. Figure 1—figure supplement 1B-SourceData-2020-07-17-FigureS1-NdeIGeno.tiff – Raw agarose gel image for Figure 1—figure supplement 1B. Figure 1—figure supplement 1C-SourceData-DKC1A386T-34.2DA9_B05_045.ab1 – Sanger sequencing file for Figure 1—figure supplement 1C of BU3 NGST DKC1 A386T mutant iPS line. Figure 1—figure supplement 1C-SourceData-WT-33.3EF6_A05_047.ab1 – Sanger sequencing file for Figure 1—figure supplement 1C of BU3 NGST wild type (WT) control iPS line. Figure 1—figure supplement 1E-SourceData-DKC1A386T-KaryotypeImageCLG-35870.pdf – Image of a representative karyotype of BU3 NGST DKC1 A386T iPS line. Figure 1—figure supplement 1E-SourceData-DKC1A386T-REPORTCLG-35870.pdf – Report of karyotype analysis for BU3 NGST DKC1 A386T iPS line. Figure 1—figure supplement 1E-SourceData-WT-KaryotypeImageCLG-35869.pdf – Image of a representative karyotype of BU3 NGST WT control iPS line. Figure 1—figure supplement 1E-SourceData-WT-REPORTCLG-35869.pdf – Report of karyotype analysis for BU3 NGST WT control iPS line. [file elife-64430-fig1-figsupp1-data1.zip › Figure1_FigureSupplement1_SourceData/Figure1-FigureSupplement1E-SourceData-DKC1A386T-KaryotypeImageCLG-35870.pdf]

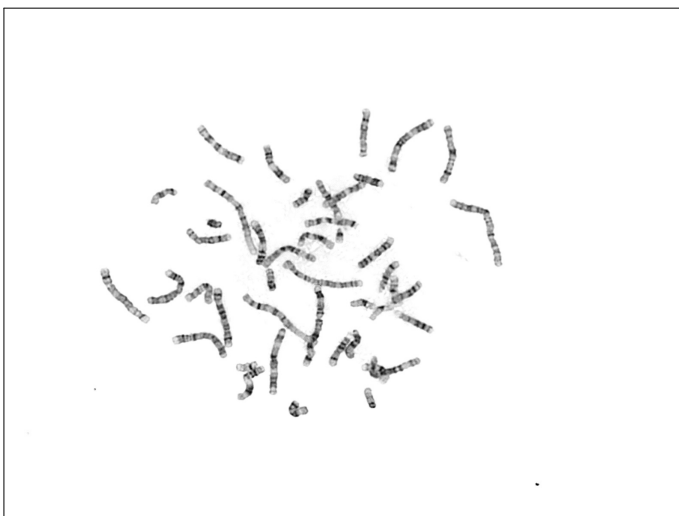

Result: 46,XY

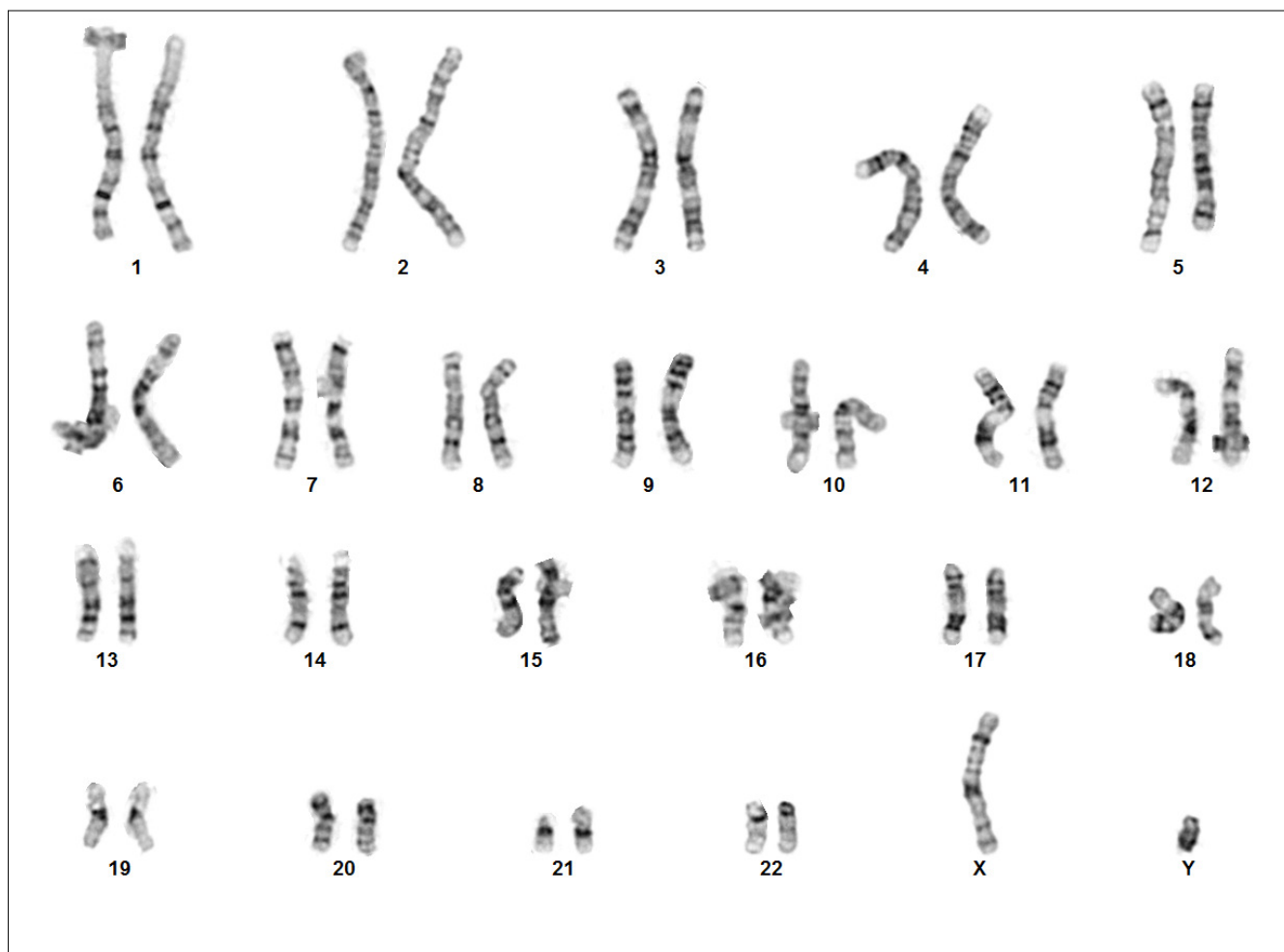

Supplement: Figure 1—figure supplement 1—source data 1. — Figure 1—figure supplement 1B-SourceData-2020-07-17-FigureS1-NdeIGeno-CroppingLabeled.tiff – Genotyping agarose gel showing area that was cropped for the Figure 1—figure supplement 1B. Figure 1—figure supplement 1B-SourceData-2020-07-17-FigureS1-NdeIGeno.tiff – Raw agarose gel image for Figure 1—figure supplement 1B. Figure 1—figure supplement 1C-SourceData-DKC1A386T-34.2DA9_B05_045.ab1 – Sanger sequencing file for Figure 1—figure supplement 1C of BU3 NGST DKC1 A386T mutant iPS line. Figure 1—figure supplement 1C-SourceData-WT-33.3EF6_A05_047.ab1 – Sanger sequencing file for Figure 1—figure supplement 1C of BU3 NGST wild type (WT) control iPS line. Figure 1—figure supplement 1E-SourceData-DKC1A386T-KaryotypeImageCLG-35870.pdf – Image of a representative karyotype of BU3 NGST DKC1 A386T iPS line. Figure 1—figure supplement 1E-SourceData-DKC1A386T-REPORTCLG-35870.pdf – Report of karyotype analysis for BU3 NGST DKC1 A386T iPS line. Figure 1—figure supplement 1E-SourceData-WT-KaryotypeImageCLG-35869.pdf – Image of a representative karyotype of BU3 NGST WT control iPS line. Figure 1—figure supplement 1E-SourceData-WT-REPORTCLG-35869.pdf – Report of karyotype analysis for BU3 NGST WT control iPS line. [file elife-64430-fig1-figsupp1-data1.zip › Figure1_FigureSupplement1_SourceData/Figure1-FigureSupplement1E-SourceData-WT-KaryotypeImageCLG-35869.pdf]

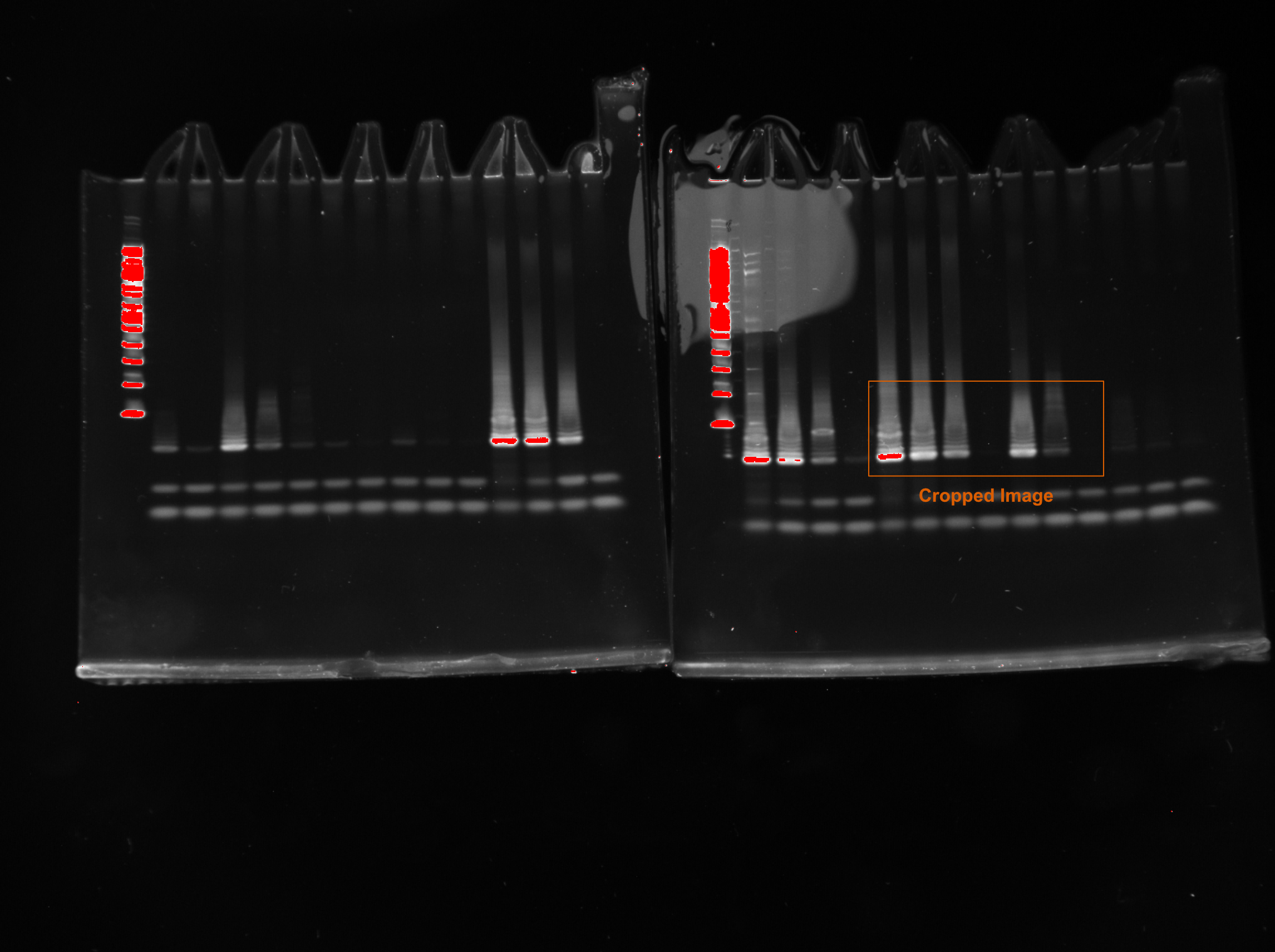

Supplement: Figure 1—figure supplement 2—source data 1. — Figure 1—figure supplement 2A-SourceData-qTRAPiPS-2018-09-06-007_qTRAP_BU3_Paired_Cell_Line.xlsx – Raw Cq (quantification cycle) data for quantitative telomeric repeat amplification protocol of different iPS lines. Figure 1—figure supplement 2B-SourceData-iPSWTDKC1A386TTRAP-2020-07-24-102Intense.tiff – Uncropped image of telomeric repeat amplification protocol (TRAP) gel for Figure 1—figure supplement 2B. Figure 1—figure supplement 2C-SourceData-2019-03-29-055TRFBU3A386T_60_60_60-Cropped.tif – Uncropped image of TRAP gel for Figure 1—figure supplement 2B showing where cropping occurred. Figure 1—figure supplement 2C-SourceData-2019-03-29-055TRFBU3A386T_60_60_60.tif – Uncropped raw image of Terminal restriction fragment (TRF blot for Figure 1—figure supplement 2C). Figure 1—figure supplement 2C-SourceData-2019-03-29-055TRFBU3A386T_60_60_60-Cropped.tiff – Uncropped raw image of TRF blot for Figure 1—figure supplement 2C showing how cropping removed the central ladder to aid in observation of iPS lines. [file elife-64430-fig1-figsupp2-data1.zip › Figure1_FigureSupplement2_SourceData/Figure1-FigureSupplement2B-SourceData-iPSWTDKC1A386TTRAP-2020-07-24-102Intense.tiff]

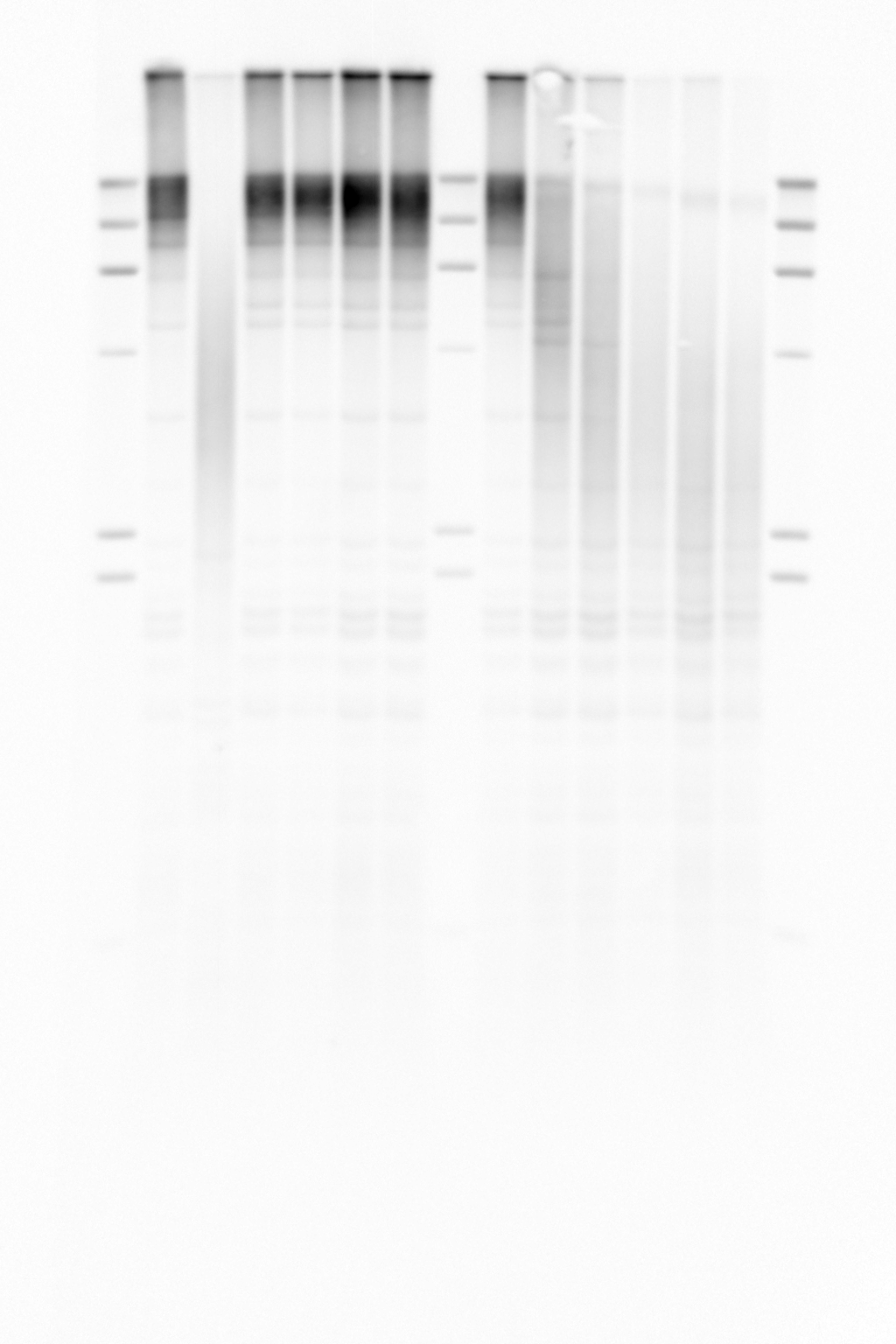

Supplement: Figure 1—figure supplement 2—source data 1. — Figure 1—figure supplement 2A-SourceData-qTRAPiPS-2018-09-06-007_qTRAP_BU3_Paired_Cell_Line.xlsx – Raw Cq (quantification cycle) data for quantitative telomeric repeat amplification protocol of different iPS lines. Figure 1—figure supplement 2B-SourceData-iPSWTDKC1A386TTRAP-2020-07-24-102Intense.tiff – Uncropped image of telomeric repeat amplification protocol (TRAP) gel for Figure 1—figure supplement 2B. Figure 1—figure supplement 2C-SourceData-2019-03-29-055TRFBU3A386T_60_60_60-Cropped.tif – Uncropped image of TRAP gel for Figure 1—figure supplement 2B showing where cropping occurred. Figure 1—figure supplement 2C-SourceData-2019-03-29-055TRFBU3A386T_60_60_60.tif – Uncropped raw image of Terminal restriction fragment (TRF blot for Figure 1—figure supplement 2C). Figure 1—figure supplement 2C-SourceData-2019-03-29-055TRFBU3A386T_60_60_60-Cropped.tiff – Uncropped raw image of TRF blot for Figure 1—figure supplement 2C showing how cropping removed the central ladder to aid in observation of iPS lines. [file elife-64430-fig1-figsupp2-data1.zip › Figure1_FigureSupplement2_SourceData/Figure1-FigureSupplement2C-SourceData-2019-03-29-055TRFBU3A386T_60_60_60.tif]

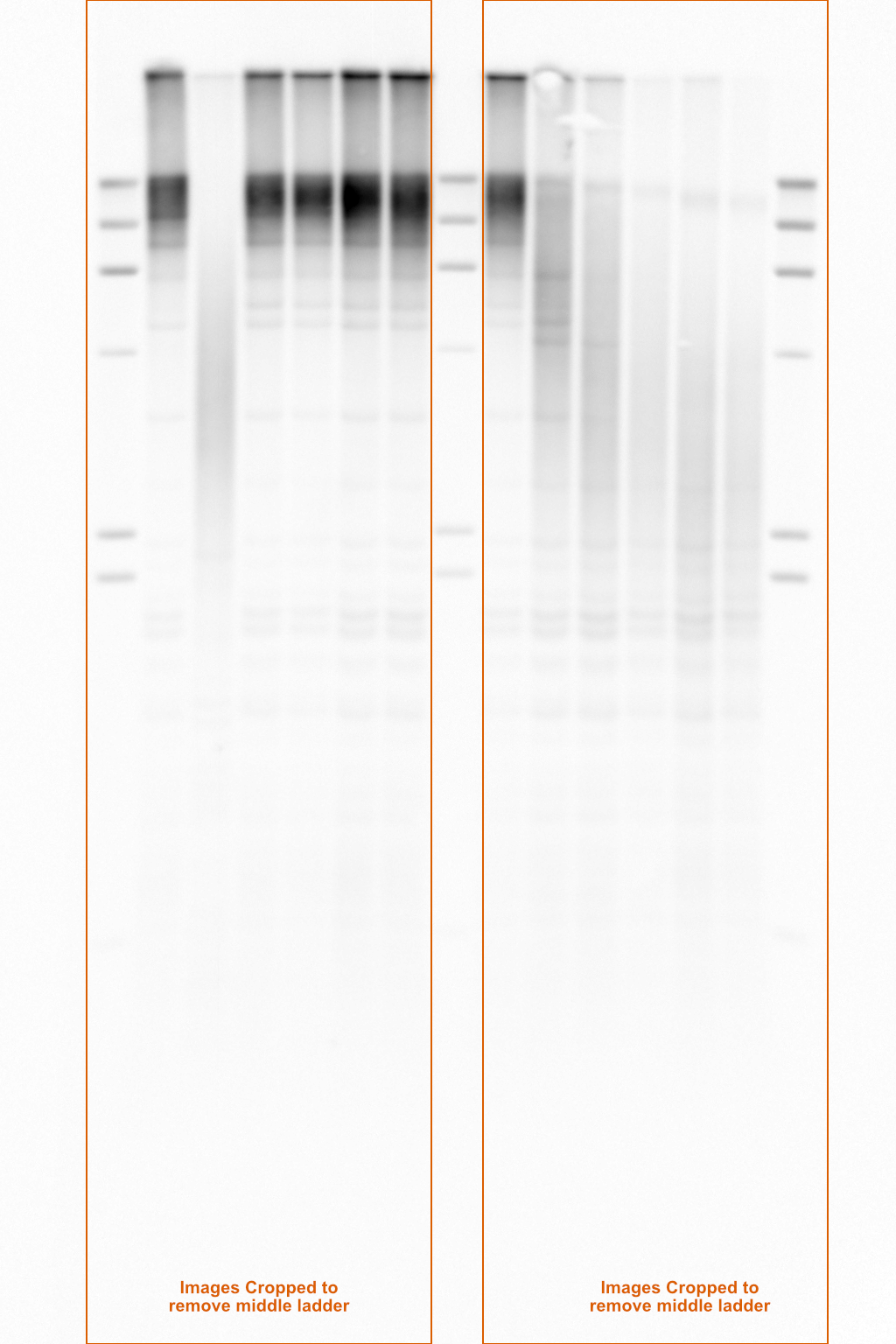

Supplement: Figure 1—figure supplement 2—source data 1. — Figure 1—figure supplement 2A-SourceData-qTRAPiPS-2018-09-06-007_qTRAP_BU3_Paired_Cell_Line.xlsx – Raw Cq (quantification cycle) data for quantitative telomeric repeat amplification protocol of different iPS lines. Figure 1—figure supplement 2B-SourceData-iPSWTDKC1A386TTRAP-2020-07-24-102Intense.tiff – Uncropped image of telomeric repeat amplification protocol (TRAP) gel for Figure 1—figure supplement 2B. Figure 1—figure supplement 2C-SourceData-2019-03-29-055TRFBU3A386T_60_60_60-Cropped.tif – Uncropped image of TRAP gel for Figure 1—figure supplement 2B showing where cropping occurred. Figure 1—figure supplement 2C-SourceData-2019-03-29-055TRFBU3A386T_60_60_60.tif – Uncropped raw image of Terminal restriction fragment (TRF blot for Figure 1—figure supplement 2C). Figure 1—figure supplement 2C-SourceData-2019-03-29-055TRFBU3A386T_60_60_60-Cropped.tiff – Uncropped raw image of TRF blot for Figure 1—figure supplement 2C showing how cropping removed the central ladder to aid in observation of iPS lines. [file elife-64430-fig1-figsupp2-data1.zip › Figure1_FigureSupplement2_SourceData/Figure1-FigureSupplement2C-SourceData-2019-03-29-055TRFBU3A386T_60_60_60-Cropped.tiff]

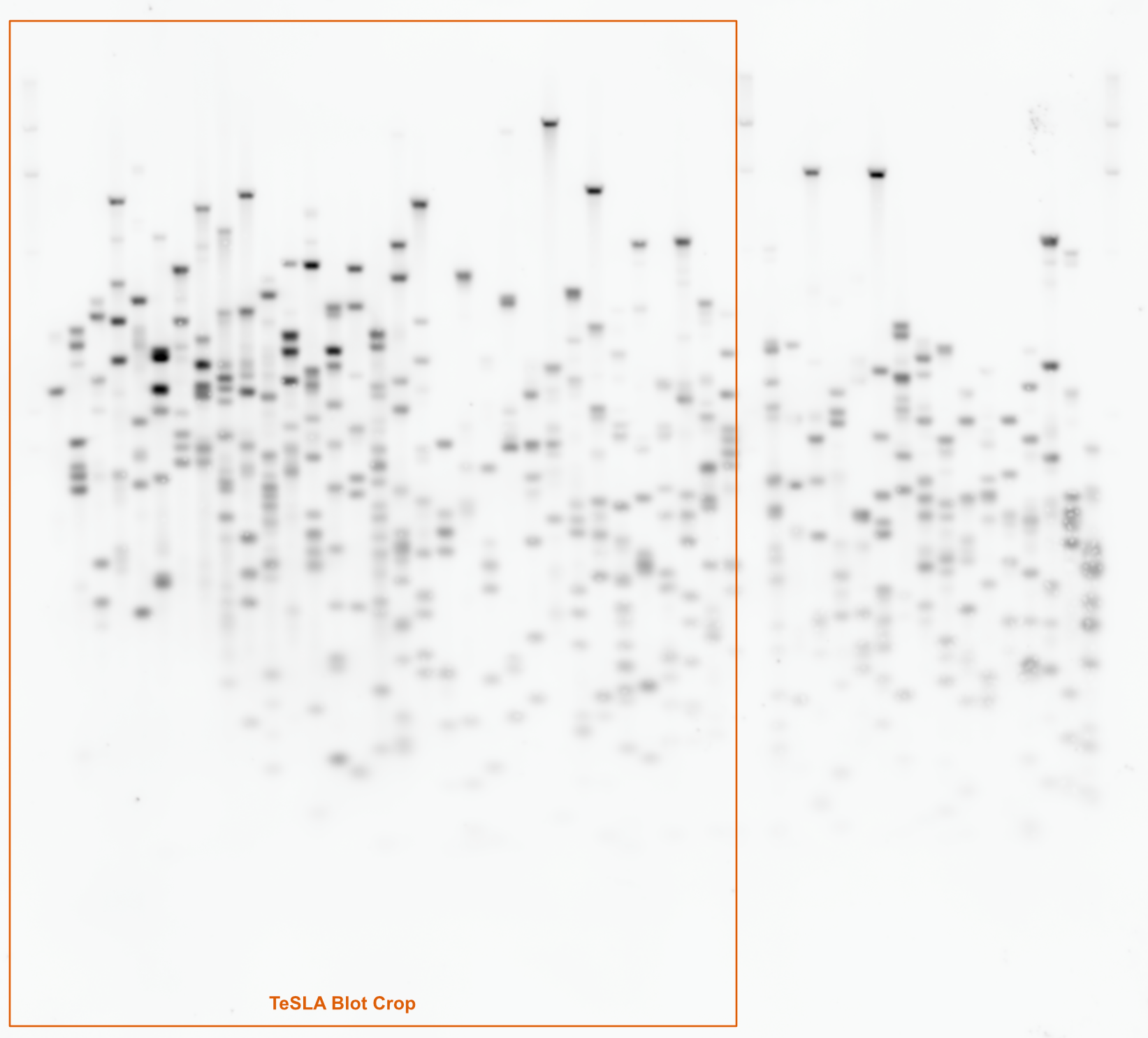

Supplement: Figure 2—source data 1. — Figure 2A-SourceData-iAT2-SenescentGeneExpression.pzfx – Raw counts from RNA-seq of induced pluripotent stem cell-derived type II alveolar epithelial cells over the differentiation specifically looking at specific senescence related genes. Figure 2B-SourceData-53BP1PositiveCounts.pzfx – Raw counts of 53BP1 positive cells along with statistics. Figure 2C-SourceData-p21PositiveCounts.pzfx – Raw counts of p21 positive cells along with statistics. Figure 2D-SourceData-TIFPhenotype.pzfx – Raw counts of telomere dysfunction induced foci positive cells along with statistics. Figure 2E,F-SourceData-2020-08-31-TeSLABlotMerged-Cropped.tiff – Uncropped raw image of TeSLA blot along the differentiation showing where the crop was placed for Figure 2E. This is the same blot as used in Figure 4—figure supplement 3B. Figure 2E,F-SourceData-2020-08-31-TeSLABlotMerged.tiff – Uncropped raw image of TeSLA blot along the differentiation showing where the crop was placed for Figure 2E. This is the same blot as used in Figure 4—figure supplement 3B. Figure 2E,F-SourceData-TeSLADKC1A386TAlongDiff.pzfx – Raw counts from TeSLA quant software analysis of cropped blot looking at telomere length along the differentiation along with statistical analysis. [file elife-64430-fig2-data1.zip › Figure2_SourceData/Figure2EF-SourceData-2020-08-31-TeSLABlotMerged-Cropped.tiff]

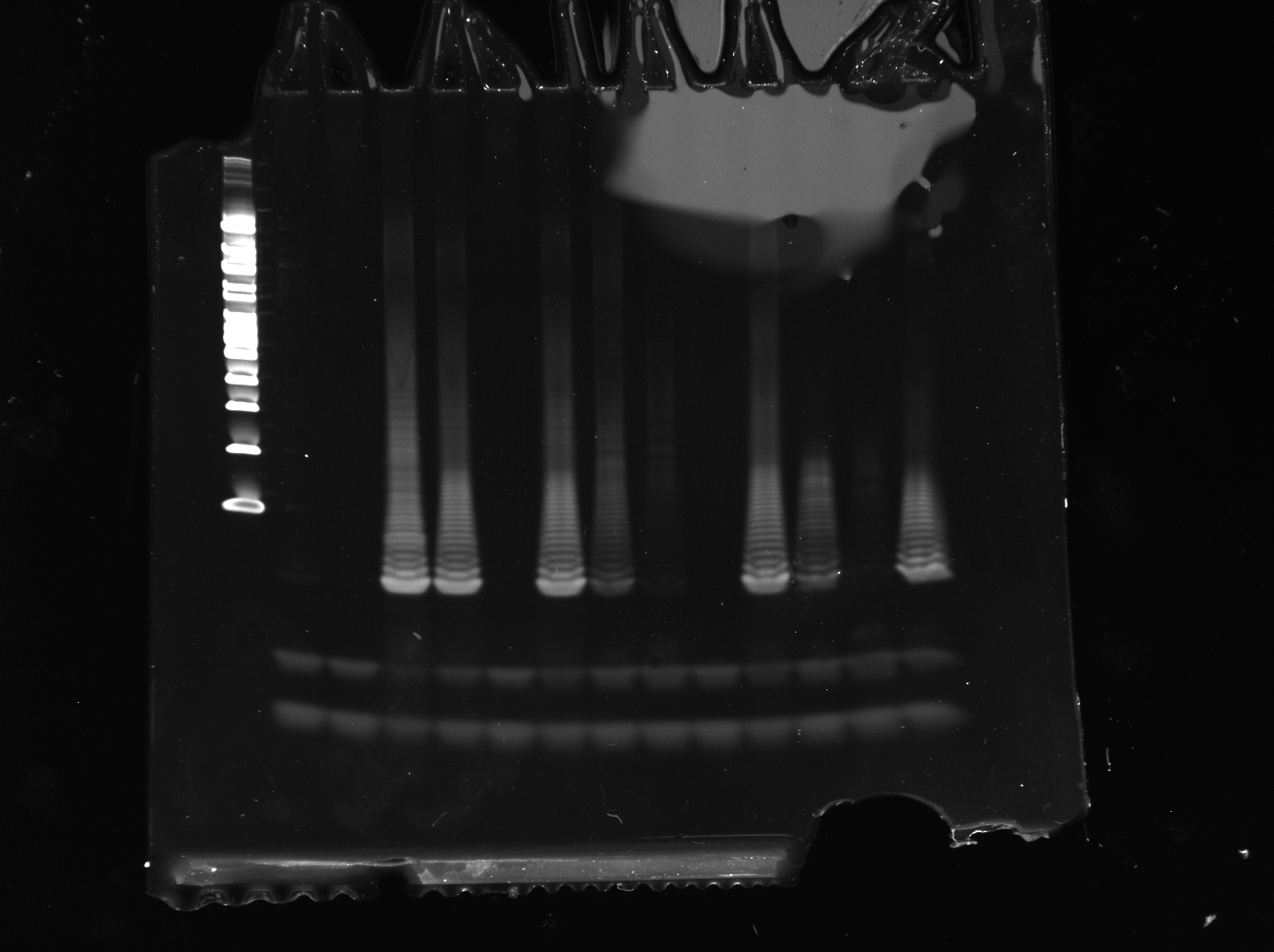

Supplement: Figure 4—source data 1. — Figure 4C-SourceData-OrganoidFormationEfficiency.pzfx – Raw counts of organoid counts normalized to number of cells input to calculate formation efficiency along with statistical tests. Figure 4D-SourceData-53BP1Rescue.pzfx – Raw counts of 53BP1 positive cells after treatment with CHIR99021 along with statistics. Figure 4E-SourceData-p21Rescue.pzfx – Raw counts of p21 positive cells after treatment with CHIR99021 along with statistics. Figure 4F-SourceData-TIFRescue.pzfx – Raw counts of telomere dysfunction induced foci positive cells after treatment with CHIR99021 along with statistics.Figure 4G-SourceData-2021-05-04-109-CK-Cropped.tiff – Raw blot of telomeric repeat amplification protocol (TRAP) assay of iPSC-derived type II alveolar epithelial (iAT2) cells treated with CHIR99021 showing area that was cropped. Figure 4G-SourceData-2021-05-04-109-CK.tif – Raw blot of TRAP assay of iAT2 cells treated with CHIR99021. Figure 4G-SourceData-2021-05-04-109-K-Cropped.tiff – Raw blot of TRAP assay of untreated iAT2 showing area that was cropped. Figure 4G-SourceData-2021-05-04-109-K.tif – Raw blot of TRAP assay of untreated iAT2. Figure 4G-SourceData-2021-05-04-109-QuantificationTRAP.xlsx – Quantifications of TRAP assay in Figure 4G. [file elife-64430-fig4-data1.zip › Figure4_SourceData/Figure4G-SourceData-2021-05-04-109-CK.tif]

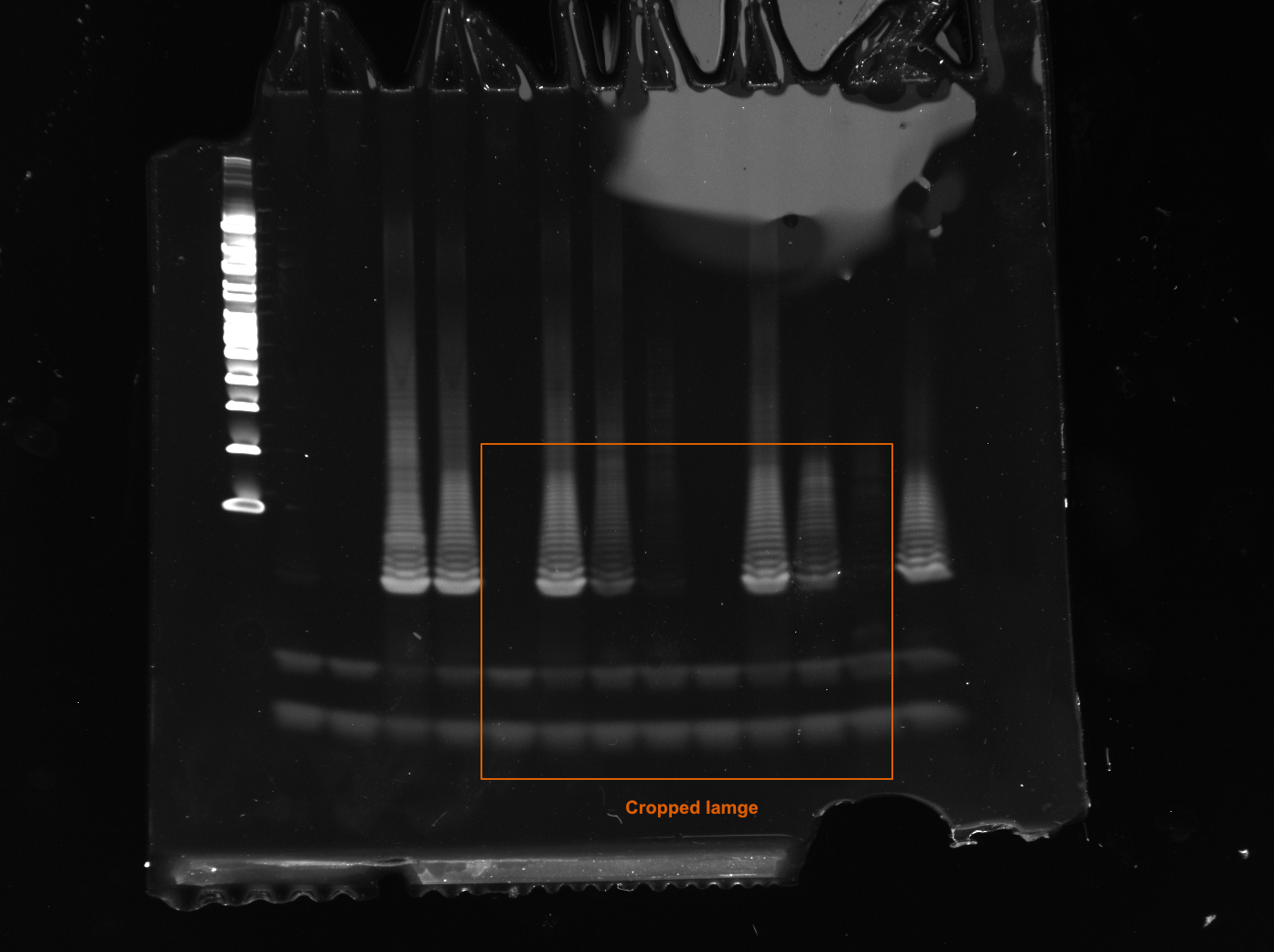

Supplement: Figure 4—source data 1. — Figure 4C-SourceData-OrganoidFormationEfficiency.pzfx – Raw counts of organoid counts normalized to number of cells input to calculate formation efficiency along with statistical tests. Figure 4D-SourceData-53BP1Rescue.pzfx – Raw counts of 53BP1 positive cells after treatment with CHIR99021 along with statistics. Figure 4E-SourceData-p21Rescue.pzfx – Raw counts of p21 positive cells after treatment with CHIR99021 along with statistics. Figure 4F-SourceData-TIFRescue.pzfx – Raw counts of telomere dysfunction induced foci positive cells after treatment with CHIR99021 along with statistics.Figure 4G-SourceData-2021-05-04-109-CK-Cropped.tiff – Raw blot of telomeric repeat amplification protocol (TRAP) assay of iPSC-derived type II alveolar epithelial (iAT2) cells treated with CHIR99021 showing area that was cropped. Figure 4G-SourceData-2021-05-04-109-CK.tif – Raw blot of TRAP assay of iAT2 cells treated with CHIR99021. Figure 4G-SourceData-2021-05-04-109-K-Cropped.tiff – Raw blot of TRAP assay of untreated iAT2 showing area that was cropped. Figure 4G-SourceData-2021-05-04-109-K.tif – Raw blot of TRAP assay of untreated iAT2. Figure 4G-SourceData-2021-05-04-109-QuantificationTRAP.xlsx – Quantifications of TRAP assay in Figure 4G. [file elife-64430-fig4-data1.zip › Figure4_SourceData/Figure4G-SourceData-2021-05-04-109-CK-Cropped.tiff]

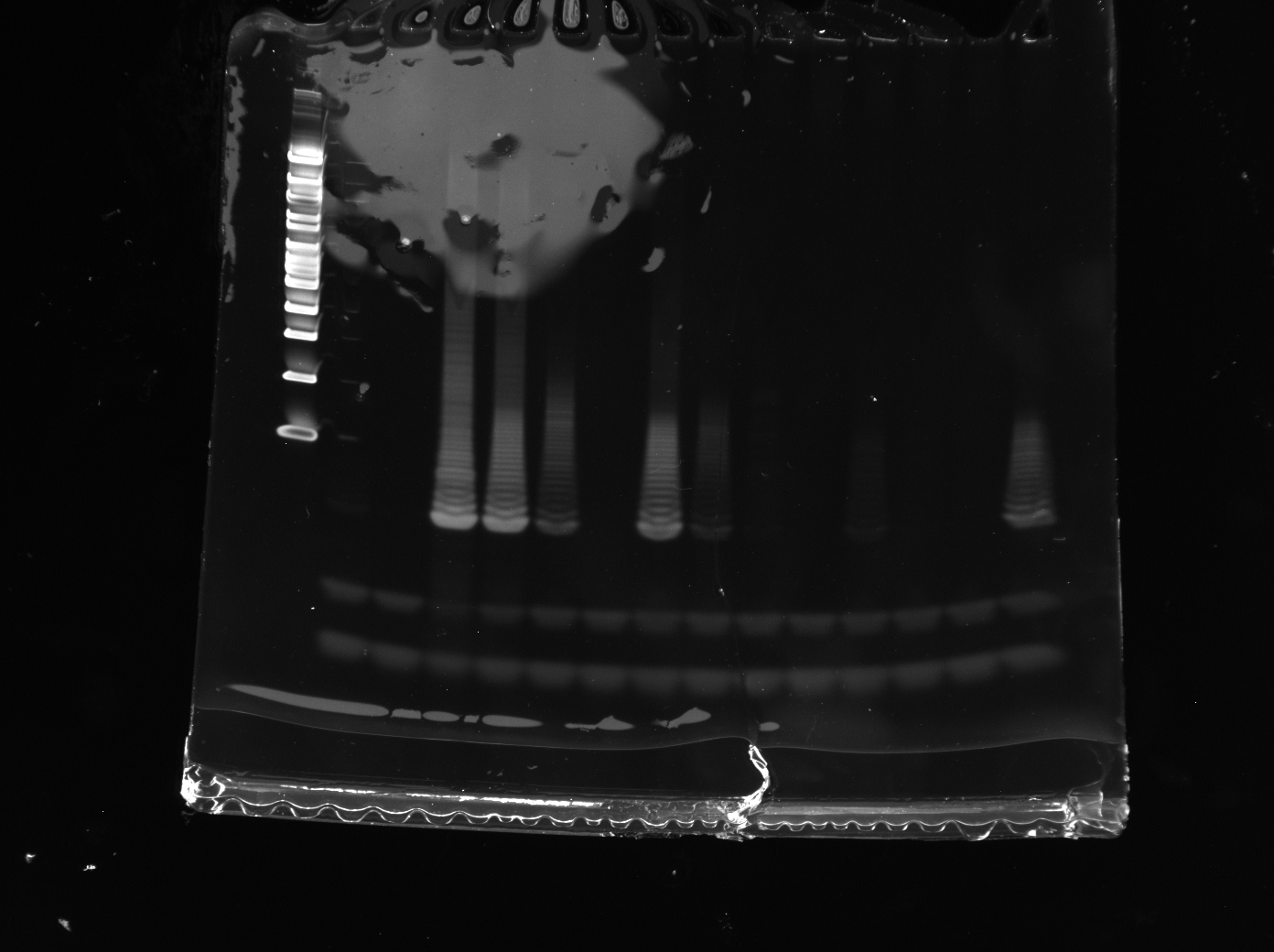

Supplement: Figure 4—source data 1. — Figure 4C-SourceData-OrganoidFormationEfficiency.pzfx – Raw counts of organoid counts normalized to number of cells input to calculate formation efficiency along with statistical tests. Figure 4D-SourceData-53BP1Rescue.pzfx – Raw counts of 53BP1 positive cells after treatment with CHIR99021 along with statistics. Figure 4E-SourceData-p21Rescue.pzfx – Raw counts of p21 positive cells after treatment with CHIR99021 along with statistics. Figure 4F-SourceData-TIFRescue.pzfx – Raw counts of telomere dysfunction induced foci positive cells after treatment with CHIR99021 along with statistics.Figure 4G-SourceData-2021-05-04-109-CK-Cropped.tiff – Raw blot of telomeric repeat amplification protocol (TRAP) assay of iPSC-derived type II alveolar epithelial (iAT2) cells treated with CHIR99021 showing area that was cropped. Figure 4G-SourceData-2021-05-04-109-CK.tif – Raw blot of TRAP assay of iAT2 cells treated with CHIR99021. Figure 4G-SourceData-2021-05-04-109-K-Cropped.tiff – Raw blot of TRAP assay of untreated iAT2 showing area that was cropped. Figure 4G-SourceData-2021-05-04-109-K.tif – Raw blot of TRAP assay of untreated iAT2. Figure 4G-SourceData-2021-05-04-109-QuantificationTRAP.xlsx – Quantifications of TRAP assay in Figure 4G. [file elife-64430-fig4-data1.zip › Figure4_SourceData/Figure4G-SourceData-2021-05-04-109-K.tif]

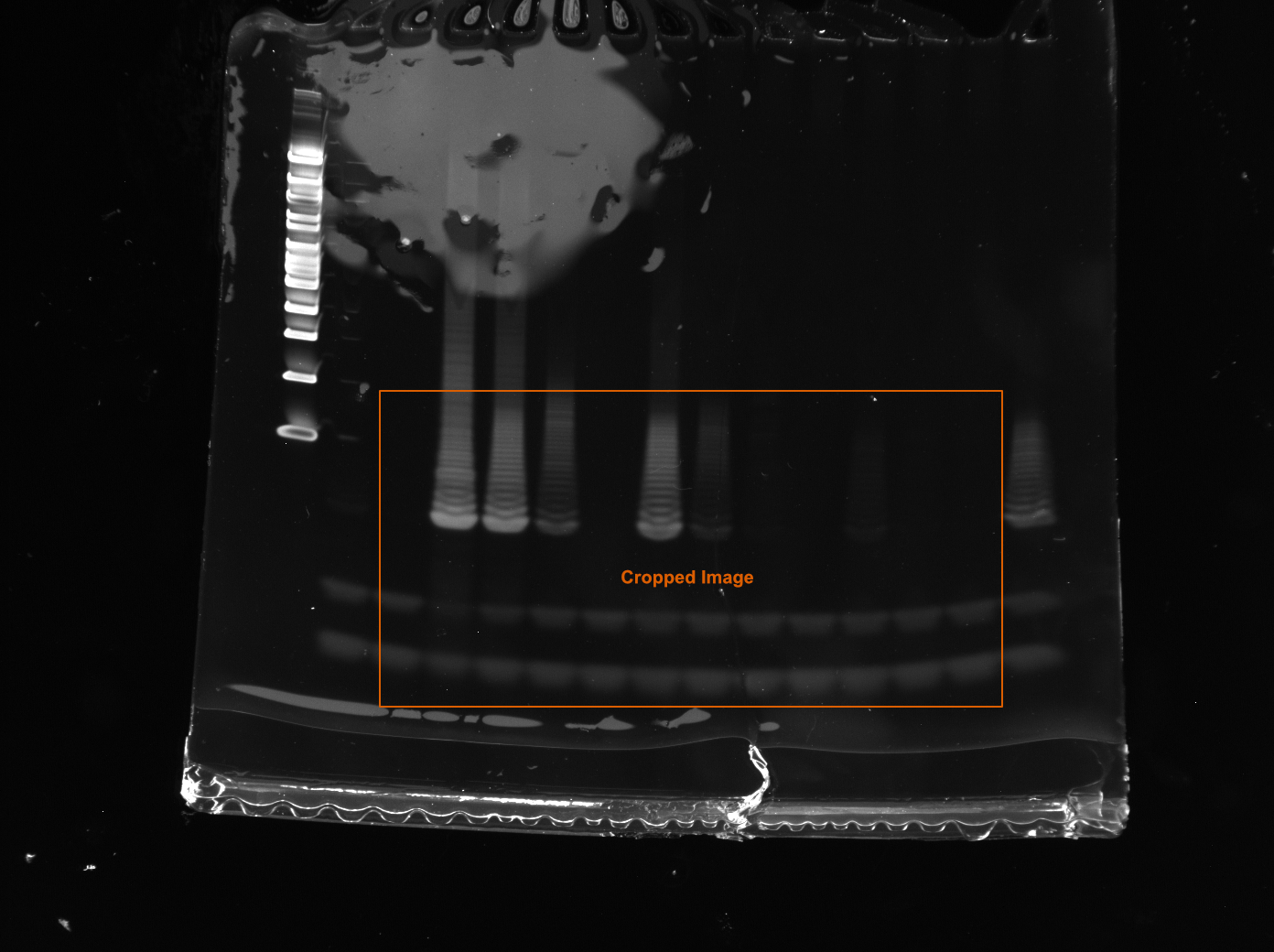

Supplement: Figure 4—source data 1. — Figure 4C-SourceData-OrganoidFormationEfficiency.pzfx – Raw counts of organoid counts normalized to number of cells input to calculate formation efficiency along with statistical tests. Figure 4D-SourceData-53BP1Rescue.pzfx – Raw counts of 53BP1 positive cells after treatment with CHIR99021 along with statistics. Figure 4E-SourceData-p21Rescue.pzfx – Raw counts of p21 positive cells after treatment with CHIR99021 along with statistics. Figure 4F-SourceData-TIFRescue.pzfx – Raw counts of telomere dysfunction induced foci positive cells after treatment with CHIR99021 along with statistics.Figure 4G-SourceData-2021-05-04-109-CK-Cropped.tiff – Raw blot of telomeric repeat amplification protocol (TRAP) assay of iPSC-derived type II alveolar epithelial (iAT2) cells treated with CHIR99021 showing area that was cropped. Figure 4G-SourceData-2021-05-04-109-CK.tif – Raw blot of TRAP assay of iAT2 cells treated with CHIR99021. Figure 4G-SourceData-2021-05-04-109-K-Cropped.tiff – Raw blot of TRAP assay of untreated iAT2 showing area that was cropped. Figure 4G-SourceData-2021-05-04-109-K.tif – Raw blot of TRAP assay of untreated iAT2. Figure 4G-SourceData-2021-05-04-109-QuantificationTRAP.xlsx – Quantifications of TRAP assay in Figure 4G. [file elife-64430-fig4-data1.zip › Figure4_SourceData/Figure4G-SourceData-2021-05-04-109-K-Cropped.tiff]

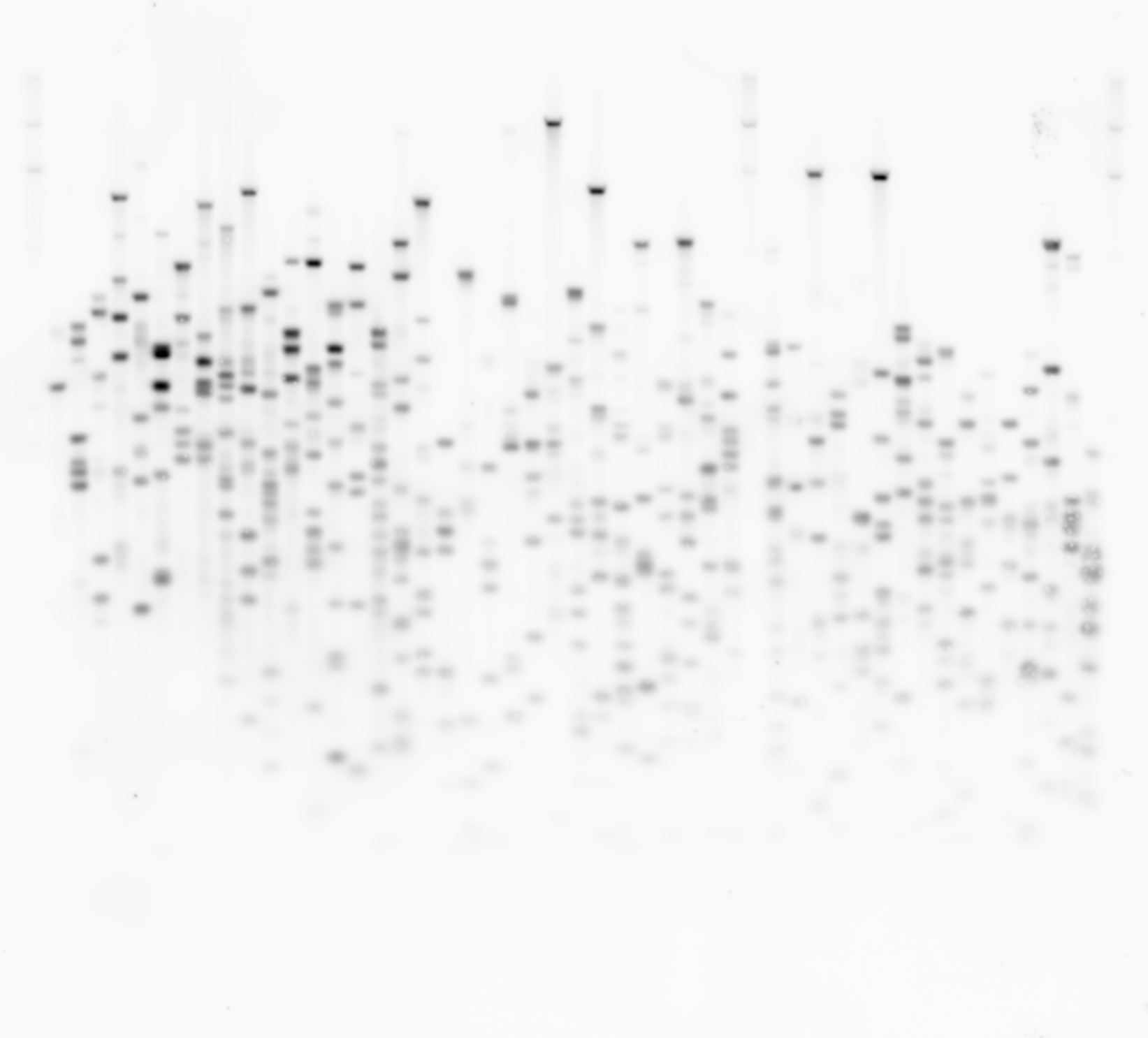

Supplement: Figure 4—figure supplement 3—source data 1. — Figure 4—figure supplement 3B-SourceData-2020-08-31-MergedTeslaDKC1A386T-Cropped – Uncropped raw image of TeSLA blot when DKC1 A386T iPSC-derived type II alveolar epithelial cells (iAT2s) were treated with CHIR99021 showing where the crop was placed for Figure 4—figure supplement 3B. This is the same blot as used in Figure 2E. Figure 4, Figure 4—figure supplement 3-SourceData-2020-08-31-MergedTeslaDKC1A386T – Uncropped raw image of TeSLA blot when DKC1 A386T iAT2s were treated with CHIR99021. This is the same blot as used in Figure 2E. Figure 4—figure supplement 3C-SourceData-DKC1A386TTesLARescue – Quantification of TeSLA using TeSLA quant software for DKC1 A386T mutant cells treated with CHIR99021. [file elife-64430-fig4-figsupp3-data1.zip › Figure4_FigureSupplement3_SourceData/Figure4-FigureSupplement3B-SourceData-2020-08-31-MergedTeslaDKC1A386T.tiff]

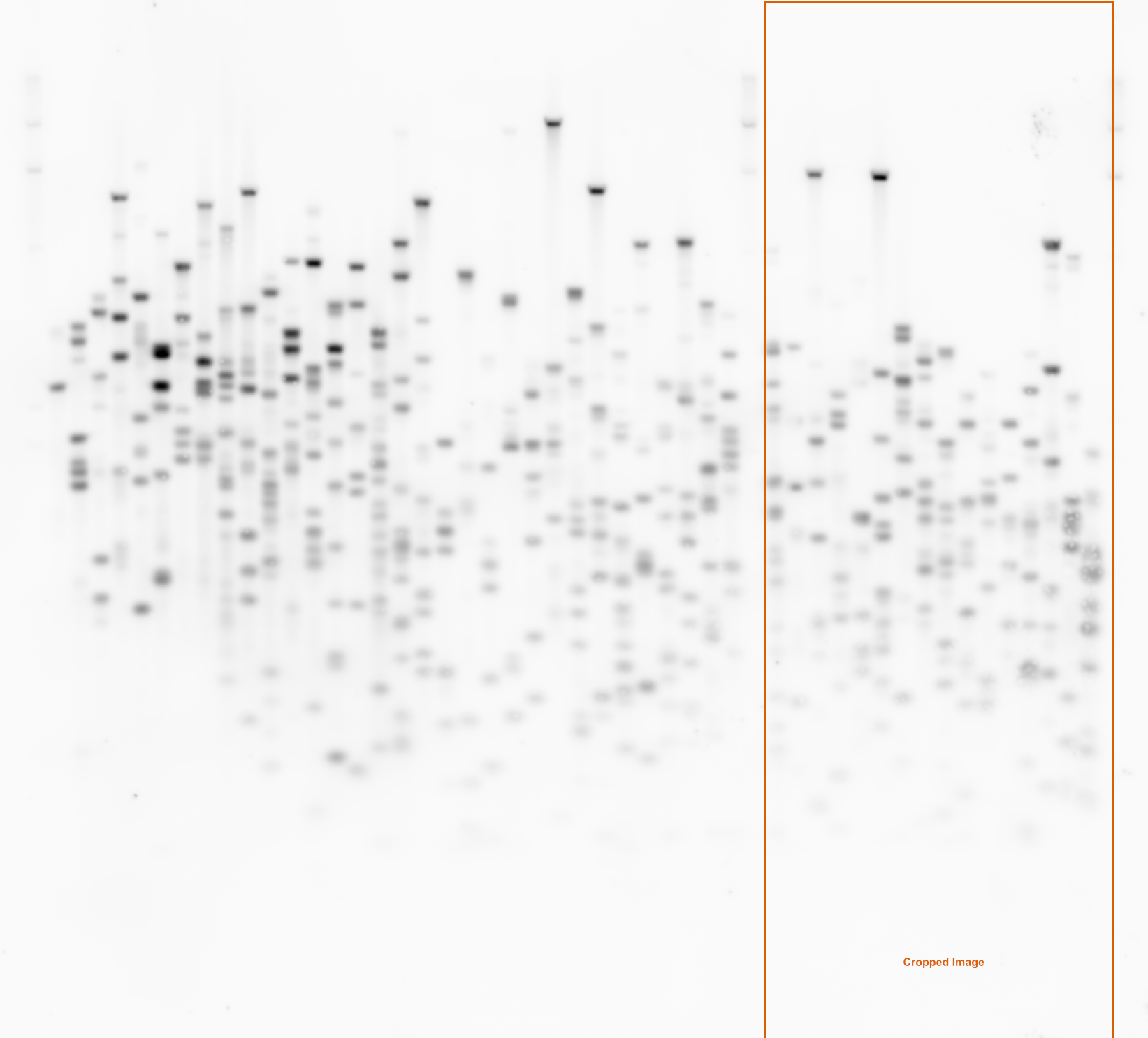

Supplement: Figure 4—figure supplement 3—source data 1. — Figure 4—figure supplement 3B-SourceData-2020-08-31-MergedTeslaDKC1A386T-Cropped – Uncropped raw image of TeSLA blot when DKC1 A386T iPSC-derived type II alveolar epithelial cells (iAT2s) were treated with CHIR99021 showing where the crop was placed for Figure 4—figure supplement 3B. This is the same blot as used in Figure 2E. Figure 4, Figure 4—figure supplement 3-SourceData-2020-08-31-MergedTeslaDKC1A386T – Uncropped raw image of TeSLA blot when DKC1 A386T iAT2s were treated with CHIR99021. This is the same blot as used in Figure 2E. Figure 4—figure supplement 3C-SourceData-DKC1A386TTesLARescue – Quantification of TeSLA using TeSLA quant software for DKC1 A386T mutant cells treated with CHIR99021. [file elife-64430-fig4-figsupp3-data1.zip › Figure4_FigureSupplement3_SourceData/Figure4-FigureSupplement3B-SourceData-2020-08-31-MergedTeslaDKC1A386T-Cropped.tiff]
